# Patient preferences for features of HER2-targeted treatment of advanced or metastatic breast cancer: a discrete-choice experiment study

**DOI:** 10.1007/s12282-022-01394-6

**Published:** 2022-09-08

**Authors:** Carol Mansfield, Willings Botha, Gerard T. Vondeling, Kathleen Klein, Kongming Wang, Jasmeet Singh, Michelle D. Hackshaw

**Affiliations:** 1 RTI Health Solutions, Research Triangle Park, NC USA; 2 RTI Health Solutions, Manchester, UK; 3 Daiichi Sankyo Europe, Munich, Germany; 4 Daiichi Sankyo Inc, Basking Ridge, NJ, USA

**Keywords:** Discrete choice, Conjoint analysis, Trade-off, Risk–benefit

## Abstract

**Background:**

We aimed to quantify patients’ benefit-risk preferences for attributes associated with human epidermal growth factor receptor 2 (HER2)-targeted breast cancer treatments and estimate minimum acceptable benefits (MABs), denominated in additional months of progression-free survival (PFS), for given treatment-related adverse events (AEs).

**Methods:**

We conducted an online discrete-choice experiment (DCE) among patients with self-reported advanced/metastatic breast cancer in the United States, United Kingdom, and Japan (*N* = 302). In a series of nine DCE questions, respondents chose between two hypothetical treatment profiles created by an experimental design. Profiles were defined by six attributes with varying levels: PFS, nausea/vomiting, diarrhea, liver function problems, risk of heart failure, and risk of serious lung damage and infections. Data were analyzed using an error component random-parameters logit model.

**Results:**

Among the attributes, patients placed the most importance on a change in PFS from 5 to 26 months; change from no diarrhea to severe diarrhea was the least important. Avoiding a 15% risk of heart failure had the largest MAB (5.8 additional months of PFS), followed by avoiding a 15% risk of serious lung damage and infections (4.6 months), possible severe liver function problems (4.2 months), severe nausea/vomiting (3.7 months), and severe diarrhea (2.3 months) compared with having none of the AEs. The relative importance of 21 additional months of PFS (increasing from 5 to 26 months) increased for women with HER2-negative disease and those with children.

**Conclusions:**

Patients valued PFS gain higher than the potential risk of AEs when deciding between hypothetical breast cancer treatments.

## Introduction

Globally, breast cancer is the most common cancer in women, with more than 2 million new cases diagnosed in 2018 and 0.5 million deaths worldwide [1]. Approximately 25–30% of breast cancers harbor a gene mutation that produces an excess of the human epidermal growth factor receptor 2 (HER2) protein [2]. HER2-positive tumors tend to be more aggressive, with an increased probability of relapse and increased risk of brain metastases, and less likely to respond to treatment compared with other breast cancer subtypes [3–5]. HER2-positive breast cancer is associated with decreases in overall survival (OS) and time in remission [6]. Several treatment options specifically for HER2*-*positive breast cancer are available, including trastuzumab, pertuzumab, the antibody–drug conjugate ado-trastuzumab emtansine (T-DM1), and, more recently, trastuzumab deruxtecan and tucatinib. Other traditional chemotherapy used to treat this patient population following T-DM1 treatment includes eribulin, capecitabine, vinorelbine, and lapatinib [7–9]. These treatments have improved progression-free survival (PFS) and OS [10]. Nevertheless, most patients with HER2-positive breast cancer eventually do not respond to these treatments [11].

New treatments for HER2-positive breast cancer that offer longer survival benefits have been approved, but these treatments have different safety profiles. Little is currently known about patient preferences for the benefit and risk attributes associated with advanced breast cancer treatments or the willingness of patients with advanced disease to take on safety risks associated with continued treatment to obtain additional PFS benefits. Using a discrete-choice experiment (DCE), we aimed to quantify patients’ benefit-risk preferences for the attributes that differentiate HER2-targeted treatments for advanced or metastatic breast cancer, estimate the relative importance of efficacy and safety attributes evaluated in clinical trials, and calculate the minimum acceptable benefit (MAB) in terms of PFS to accept the risk of a given side effect. The sample included women with self-reported stage III/IV breast cancer from the United States (US), the United Kingdom (UK), and Japan. We also evaluated differences in preferences between subgroups defined by disease stage, prior treatment experience, HER2 status, whether the respondent has children, whether the respondent has ever worked in the medical field, and the respondent’s country of residence.

## Materials and methods

### Survey development

A DCE survey was administered to patients with self-reported stage III/IV breast cancer in the US, the UK, and Japan. Development of the survey followed good research practice guidelines [12–14] and Center for Devices and Radiological Health guidelines [15].

**Fig. 1:**
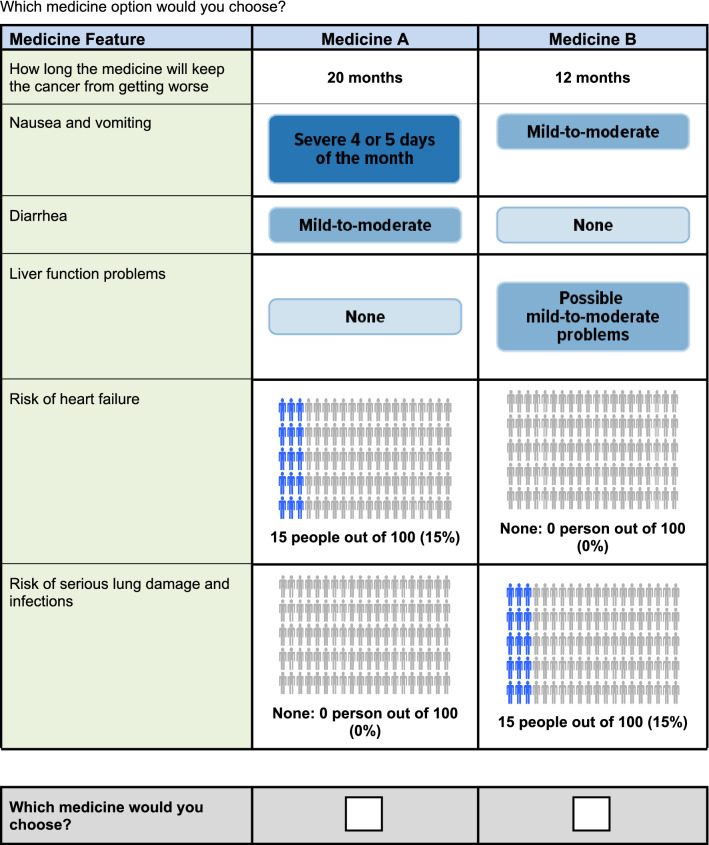
Example discrete-choice experiment question

The survey instrument asked respondents to assume that, after visiting their doctor for a checkup, their doctor had told the respondent that their cancer was getting worse and they needed to start a new treatment. Respondents answered a series of nine DCE questions. Each question offered a choice between two hypothetical treatment profiles, Medicine A or Medicine B. The hypothetical treatments in the choice questions were defined by six attributes with a range of varying levels (Table 1). Figure 1 presents an example DCE question in which each hypothetical treatment profile reflected one of the levels for each attribute. Previous DCEs conducted in oncology identified the importance to patients of efficacy, including OS and PFS, mild-to-moderate adverse events (AEs) that affect daily quality of life, and the risk of severe AEs [16]. Consistent with the purpose of this study, attributes were selected to characterize the key benefit as well as important AEs associated with HER2-targeted therapies for advanced or metastatic breast cancer, including newer treatments and AEs that affect daily quality of life. The ranges of attribute levels were based on available data associated with approved and investigational HER2-targeted therapies and were described to correspond to the risk of serious AEs and to grades defined by the Common Terminology Criteria for Adverse Events [17–23]. Efficacy was described in terms of PFS, rather than OS, because PFS data were available for all treatments of interest at the time the survey was designed [18–23]. In addition, the survey instrument contained questions on the respondent’s disease and treatment experience, demographic questions, and questions to assess respondent comprehension of the treatment attributes and DCE questions. The Metastatic Breast Cancer Alliance, a patient advocacy group, reviewed and provided input on the study protocol. Before the survey was administered to the study samples, the survey instrument was qualitatively pretested in a series of telephone interviews, each lasting approximately 60 min, with 14 eligible participants in the US, 5 in the UK, and 5 in Japan. The pretests confirmed that the survey text and questions were easily understood and the attributes and levels were relevant and appropriately described.

**Table 1:** Attributes and levels for the discrete-choice experiment survey

Attribute	Patient-friendly label and description	Levels
Progression-free survival (PFS)	Label: How long the medicine will keep the cancer from getting worse Description: An important goal of cancer medicines is to increase the length of time during and after the treatment that a patient lives with the cancer, but the cancer does not get worse (the cancer does not start growing again) Later in this survey, we will ask you to think about how long different cancer medicines may keep the cancer from getting worse (keep the cancer stable). We will show you choices between different medicines that keep the cancer from getting worse for between 5 and 26 months. If the cancer starts to grow or progress again, your doctor would talk to you about trying a different medicine	26 months 20 months 12 months 5 months
Nausea and vomiting^a^	Label: Nausea and vomiting Description: Some cancer medicines can cause stomach and digestion problems including nausea and vomiting Later in the survey, we will ask you to think about the effect of cancer medicines on nausea and vomiting you may have. There are three possibilities: None: The medicine does not cause any nausea and vomiting Mild-to-moderate: You may lose your appetite and eat less than normal. You may vomit 1–5 times per day. The nausea and vomiting may limit your ability to do your normal activities. Often the problems can be helped with over-the-counter medicine Severe 4–5 days each month: For 4–5 days each month you will have severe symptoms. You may not have any appetite. You may vomit 6 times or more a day. In some cases, the problems may be severe enough to require emergency treatment and may require you to stay in the hospital overnight. In rare cases, these problems may be life-threatening. You will have mild-to-moderate symptoms most of the month	None Mild-to-moderate Severe 4 or 5 days of the month
Diarrhea^a^	Label: Diarrhea Description: Some cancer medicines can cause diarrhea (loose or watery stool). If you already have frequent diarrhea, the medicine will not make it better, but it could make the diarrhea worse Later in the survey, we will ask you to think about the effect of cancer medicines on how much diarrhea you may have. There are three possibilities: None: The medicine does not cause diarrhea Mild-to-moderate: You may have 1 to 6 loose stools per day. The diarrhea may limit your ability to go places without an easily accessible bathroom. Often the problem can be managed with over-the-counter medicine Severe 4–5 days each month: For 4–5 days each month you will have severe symptoms. You may have 7 or more loose stools per day. You sometimes cannot make it to the toilet in time. You may experience extreme fluid loss (dehydration) and need intravenous (IV) fluids. In some cases, the problems may be severe enough to require you to stay in the hospital overnight. In rare cases, the problems may be life-threatening. You will have mild-to-moderate symptoms most of the month	None Mild-to-moderate Severe 4 or 5 days of the month
Liver function problems	Label: Liver function problems Description: Some cancer medicines can injure your liver function. The liver does several important things in your body, including filtering toxic substances out of your body and producing proteins your body needs. Severe liver injury may lead to hepatitis, an inflammation of your liver While you are taking the breast cancer medicine, your doctor will monitor your liver function using blood tests. If tests indicate that the medicine is causing injury to your liver function, your doctor may change the medicine you take Later in this survey, we will ask you to think about breast cancer medicines that may result in different levels of liver damage. There are three possibilities: None: The medicine does not affect your liver Possible mild-to-moderate problems: Studies have found that the medicine may affect your liver. If the medicine affects your liver, the medicine will cause mild-to-moderate injury to your liver function. The liver should heal on its own. If you have mild problems, you may not experience any symptoms. If you develop moderate symptoms, you may feel tired, and it may be hard for you to do some of your normal activities. Your skin, eyes, areas around your eyes and mouth may become yellow, and you may have noticeable abdominal pain Possible severe problems: Studies have found that in 1% of patients or less, the medicine has caused severe injuries to liver function, in addition to mild-to-moderate problems. If there is severe injury to liver functioning, you may have severe fatigue, abdominal pain, severe nausea and vomiting, and problems with bleeding that does not stop after a few minutes. You will need to get medical treatment for severe liver problems. In some cases, the problems may be severe enough to require emergency treatment and may require you to stay in the hospital overnight. In rare cases, these problems may be life-threatening	None Possible mild-to-moderate problems Possible severe problems
Risk of heart failure	Label: Risk of heart failure Description: Some cancer medicines can cause heart failure. Heart failure is different from a heart attack. Heart failure does not mean the heart stops working. Heart failure means that the heart does not work as well and blood moves through the heart and body at a slower rate People who have heart failure may experience shortness of breath, fatigue and weakness, swelling, rapid or irregular heartbeat, or other symptoms. Heart failure may cause complications such as kidney or liver damage, heart valve problems, or heart rhythm problems. In some cases, heart failure may be life-threatening Later in this survey, we will ask you to think about cancer medicines that have different risks of heart failure that range from no risk to a 15% risk	None: 0 people out of 100 (0%) 1 person out of 100 (1%) 15 people out of 100 (15%)
Risk of serious lung damage and infections	Label: Risk of serious lung damage and infections Description: Some cancer medicines increase the risk of developing lung damage that can lead to permanent shortness of breath where you feel like you cannot catch your breath. The medicine may cause scarring or inflammation in your lungs that make your lungs stiffer, which can make it harder to breathe The problem may require hospitalization. In some cases, the lung damage and infections may be life-threatening. The lung damage can be permanent and may get worse over time. The symptoms of serious lung problems include new or worsening cough, trouble breathing, fatigue, fever, new or worsening shortness of breath or other breathing issues. People who experience these side effects need to be evaluated by a doctor for potential problems with their lungs Later in the survey, we will ask you to think about cancer medicines with different risks of lung damage that range from no risk to a 15% risk	None: 0 people out of 100 (0%) 5 people out of 100 (5%) 15 people out of 100 (15%)

*CTCAE* common terminology criteria for adverse events, *IV* intravenous, *PFS* progression-free survival

^a^Side effects or adverse events were graded according to the CTCAE, v5.0, published on 27 November 2017, where the definition of mild corresponds to grade 1, moderate corresponds to grade 2, and severe corresponds to grades 3–5

The profiles were experimentally designed to have statistical properties that allowed estimation of the main-effect preference weights of interest using a random-parameters logit (RPL) model [14]. The experimental design was generated in SAS 9.4 software (SAS Institute Inc.; Cary, North Carolina) using a D-optimal algorithm to construct a fractional factorial experimental design [24, 25]. The experimental design included 72 DCE questions, which were used to create eight blocks of nine DCE questions each. Respondents were randomly assigned to one block of nine questions.

A survey was considered complete and was included in the final sample for analysis if the respondent answered at least one DCE question. Respondents were excluded if they did not show variability in their answers to the DCE choice questions or if they completed the survey too quickly (in < 6 min). Missing data on DCE questions and respondents’ demographics were not included in the DCE or subgroup models.

### Study population

Global Perspectives, an international market research firm with a speciality in healthcare, invited potential respondents in the US, the UK, and Japan via email to participate in the online survey instruments through Global Perspectives’ web-based partner panels. A quota sample of 200 respondents in the US, 50 in the UK, and 50 in Japan was targeted. Eligible individuals had a self-reported physician diagnosis of advanced breast cancer (stage III or IV), were aged ≥ 18 years, and were able to read and understand the language of the study country. All respondents provided electronic informed consent.

### Statistical analyses

The DCE data for the US, the UK, and Japan were pooled and analyzed using an error component (EC) RPL model following good research practices [14]. Before pooling the data, RPL models were estimated for each country separately, but the small sample size for the UK prevented the model from converging and that for Japan precluded drawing conclusions because of wide confidence intervals. Preliminary models suggested that the US and UK respondents had similar preferences, but that Japanese respondents might place less relative importance on an increase in months of PFS. Using the approach of Hensher et al. [26], the final EC RPL model included a term equal to 1 if the data were from Japan and equal to 0 if the data were from the US and UK that was interacted with an effects-coded alternative dummy variable that was equal to 1 if the observation was associated with the first medicine alternative presented in the DCE question. The EC RPL model helps control for potential differences in preferences between Japan and the US and UK. The EC RPL model related the choices respondents made in the DCE survey to the differences in the attribute levels across the alternatives in each choice question [14]. All attribute levels were effects coded [27].

The model yields a set of relative preference weights for the attribute levels. Relative preference weights are a measure of the relative effect of an attribute level on the utility or preference for a hypothetical treatment. A Wald ${\chi }^{2}$ test was used to determine the statistical significance of differences between adjacent attribute levels. The conditional relative importance of each attribute, or the maximum change in utility achievable with that attribute relative to all other included attributes, was calculated as the difference between the EC RPL preference weights for the most- and least-preferred levels for that attribute. In other words, conditional relative importance indicates the overall relative importance of the attribute over the range of attribute levels included in the survey. Estimates from the EC RPL model were also used to calculate the MAB as additional months of PFS required for respondents to accept worsening in the levels of each of the AEs included in the survey.

To test for observable characteristics that may be systematically associated with differences in preferences, we explored preferences among subgroups defined by breast cancer stage, prior breast cancer treatment experience, HER2 status, whether respondents had children, employment in the medical field, and the respondent’s country of residence. For each mutually exclusive set of subgroups, we created a dummy variable that was equal to 1 if the respondent belonged to the subgroup and interacted the dummy variable with each of the variables for the attribute levels in the model. The parameter on each of these interaction terms can be interpreted as the difference between the two mutually exclusive subgroups. Separate subgroup models were estimated using the EC RPL model, and differences in preferences between subgroups were tested through a log-likelihood ${\chi }^{2}$ test of joint statistical significance of all the interaction terms (*P* < 0.05).

## Results

### Respondent characteristics

The final sample (Table 2) included 302 respondents (US, 200; UK, 52; Japan, 50) who met the inclusion criteria, provided informed consent, and whose surveys were considered complete. The mean patient age was 48 years; approximately 60% had stage IV breast cancer, 40% were HER2 positive, and 76% had children. Approximately 87% of the respondents were currently receiving treatment for their breast cancer; approximately 78% had received treatment for breast cancer in the past. The most common treatments differed by country.

**Table 2:** Demographic and disease characteristics of respondents (*N* = 302)

Characteristic	US (*n* = 200)	UK (*n* = 52)	Japan (*n* = 50)	All respondents (*N* = 302)
Age, mean (SD), years	49.3 (12.0)	46.0 (9.4)	42.5 (9.9)	47.6 (11.5)
Stage of breast cancer, *n* (%)				
Stage 3	65 (32.5)	36 (69.2)	21 (42.0)	122 (40.4)
Stage 4	135 (67.5)	16 (30.8)	29 (58.0)	180 (59.6)
Time since diagnosis, *n* (%)				
Less than 1 year ago	14 (7.0)	8 (15.4)	6 (12.0)	28 (9.3)
1–5 years ago	110 (55.0)	30 (57.7)	34 (68.0)	174 (57.6)
6–10 years ago	50 (25.0)	11 (21.2)	10 (20.0)	71 (23.5)
11–20 years ago	20 (10.0)	3 (5.8)	0	23 (7.6)
More than 20 years ago	6 (3.0)	0	0	6 (2.0)
HER2 status, *n* (%)				
HER2 positive	87 (43.5)	13 (25.0)	22 (44.0)	122 (40.4)
HER2 negative	99 (49.5)	32 (61.5)	21 (42.0)	152 (50.3)
The doctor hasn’t told me about this	10 (5.0)	4 (7.7)	4 (8.0)	18 (6.0)
Don’t know or not sure	4 (2.0)	3 (5.8)	3 (6.0)	10 (3.3)
Currently receiving treatment, *n* (%)				
Yes	184 (92.0)	36 (69.2)	44 (88.0)	264 (87.4)
No	16 (8.0)	16 (30.8)	6 (12.0)	38 (12.6)
Received treatment in the past, *n* (%)				
Yes	156 (78.0)	41 (78.8)	39 (78.0)	236 (78.1)
No	44 (22.0)	11 (21.2)	11 (22.0)	66 (21.9)
Most common current treatments ^a^				
Herceptin (Trastuzumab)	44 (23.9%)	6 (16.7%)	16 (36.4%)	–
Tykerb (Lapatinib)	5 (2.7%)	3 (8.3%)	11 (25.0%)	–
Ibrance (Palbociclib)	44 (23.9%)	7 (19.4%)	2 (4.5%)	–
Has children, *n* (%)				
Yes	153 (76.5)	43 (82.7)	34 (68.0)	230 (76.2)
No	47 (23.5)	9 (17.3)	16 (32.0)	72 (23.8)
Have you ever worked in the medical field?, *n* (%)				
Yes	32 (16.0)	7 (13.5)	14 (28.0)	53 (17.5)
No	168 (84.0)	45 (86.5)	36 (72.0)	249 (82.5)

*HER2* human epidermal growth factor receptor 2, *SD* standard deviation, *UK* United Kingdom, *US* United States

^a^Other most current treatments included letrozole (38 [20.7%] for US, 4 [11.1%] for UK, and 9 [20.5%] for Japan) and eribulin (12 [6.5%] for US, 3 [8.3%] for UK, and 10 [22.7%] for Japan)

### Preference weights and conditional relative importance

**Fig. 2:**
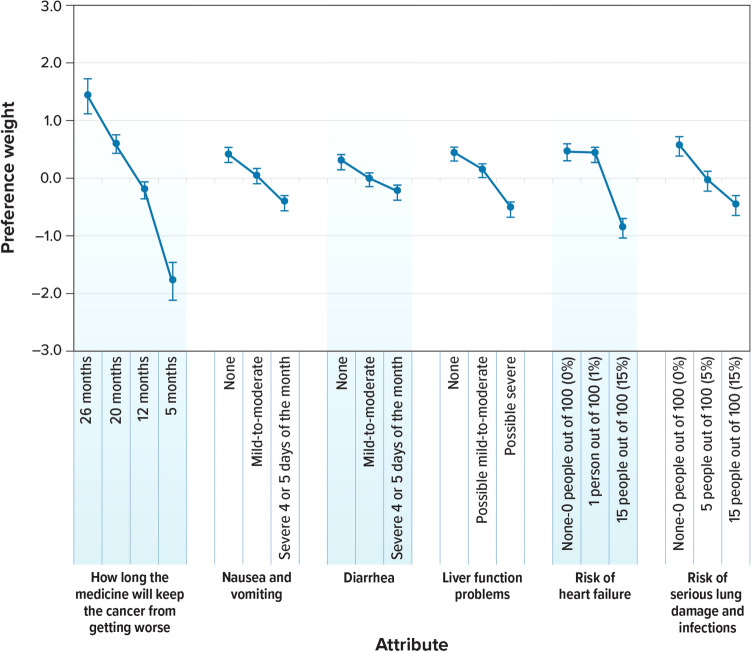
Attribute preference weights for respondents (*N* = 302). Note: The vertical bars surrounding each mean preference weight denote the 95% confidence interval of the point estimate (preference weights computed by the delta method for the level omitted in estimation for each attribute)

Preferences for attribute levels were ordered as expected, with better levels being preferred to worse levels. Figure 2 shows the normalized mean preference weight estimates for each attribute level. The change in utility associated with a change in the level of each attribute is represented by the vertical difference between the preference weights for those levels; larger differences between preference weights indicate that respondents viewed the change as having a relatively greater effect on overall utility. Most of the levels within each attribute were statistically different from one another (*P* < 0.05), except for the difference between a 0% and 1% risk of heart failure.

**Fig. 3:**
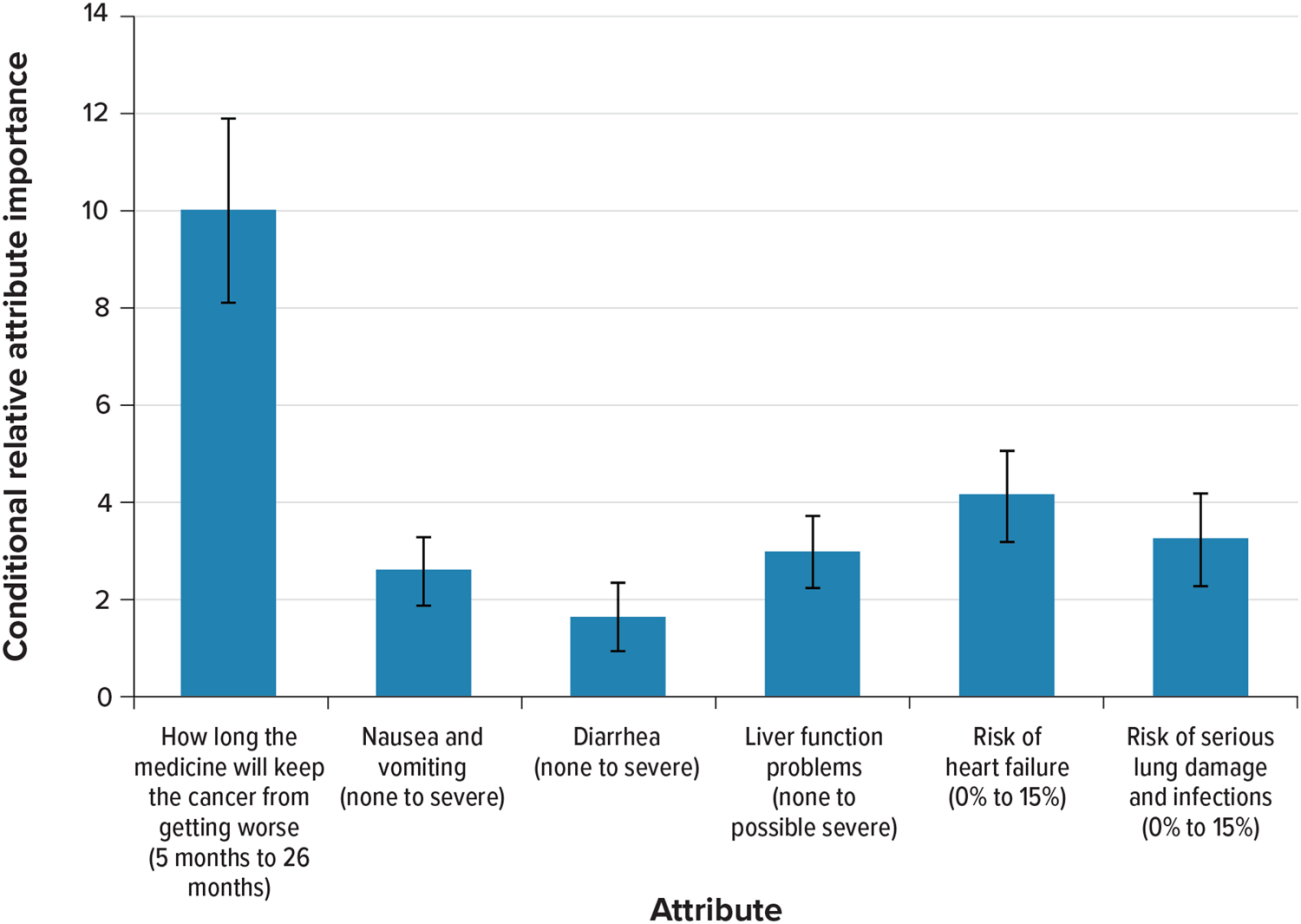
Conditional relative attribute importance for respondents (*N* = 302). *PFS* progression-free survival. Note: The vertical bars surrounding each bar denote the 95% confidence interval (computed by the delta method). The bars are scaled such that PFS is set to 10, and the conditional importance of each of the other attributes is scaled relative to the conditional importance of PFS

Figure 3 shows the conditional relative importance of changing each attribute from the least-preferred to the most-preferred level, scaled such that the change in PFS is set to 10. Over the ranges presented in the survey, patients placed the most importance on a change in PFS from 5 to 26 months, and the utility of the 21-month change in PFS was estimated to be statistically significantly higher than the changes in other attributes presented in the survey. This was followed by, in order, a 15% reduction in risk of heart failure, a 15% reduction in the risk of serious lung damage and infections, avoiding the possibility of severe liver function problems, avoiding severe nausea and vomiting, and avoiding severe diarrhea. The reduction in the risk of heart failure, risk of serious lung damage and infections, possibility of liver function problems, and avoiding severe nausea and vomiting were of approximately similar relative importance while avoiding severe diarrhea was statistically significantly less important than the reduction in the risk of all side effects.

### Minimum acceptable benefit

**Fig. 4:**
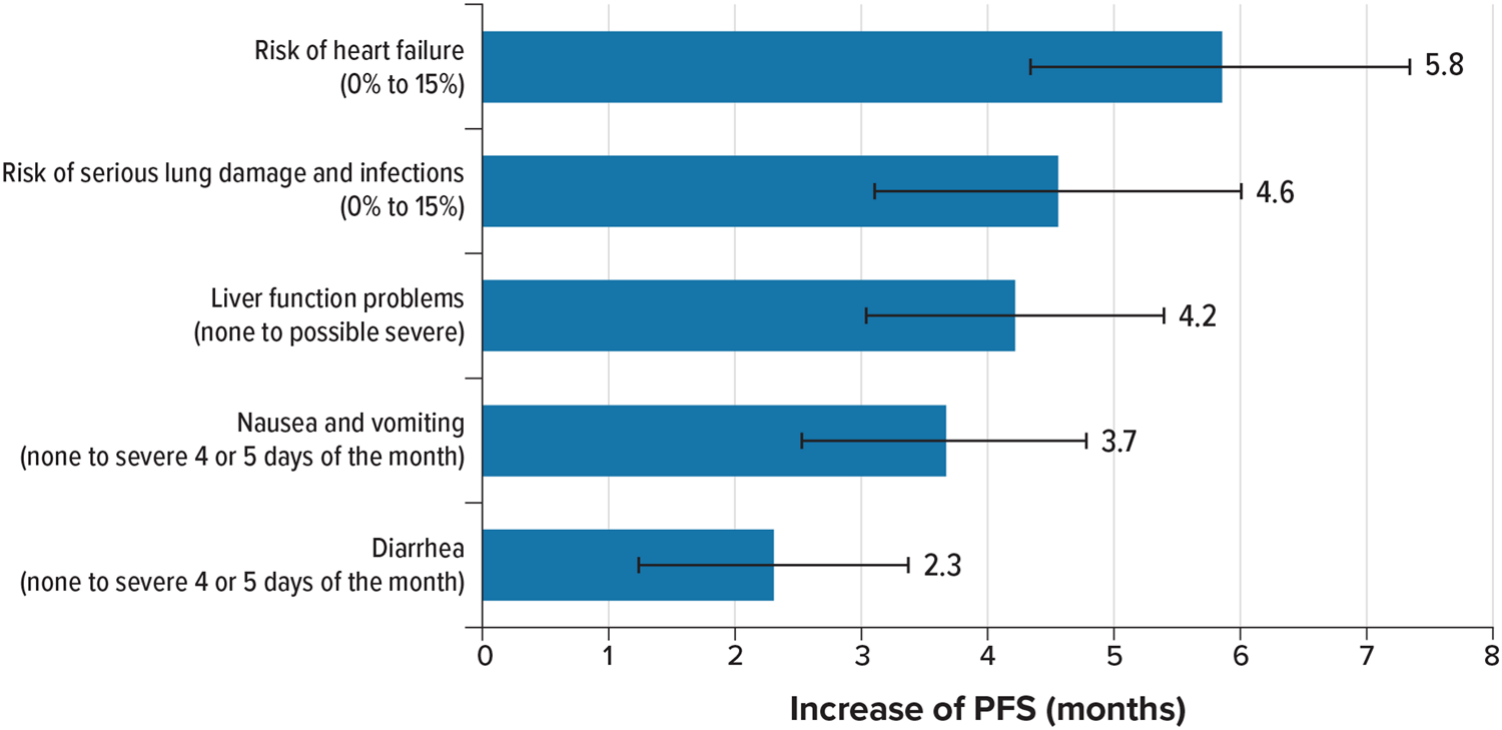
Minimum acceptable benefit in months of progression-free survival for a change in adverse event severity or risk (*N* = 302). *PFS* progression-free survival. Note: The horizontal bars surrounding each bar denote the 95% confidence interval (computed by the delta method)

MABs, shown in Fig. 4, provide another way to quantify the relative importance of changes from one level of an attribute to another. Specifically, the MAB is the number of months of PFS that would offset a change in another attribute. Accepting a 15% risk of heart failure required the largest MAB in additional months of PFS. Specifically, to accept a 15% risk of heart failure compared with 0% or 1%, respondents would require approximately a 5.8- or 5.6-month increase in PFS, respectively. The next largest MAB was an increase of approximately 4.6 months of PFS for moving from no risk of serious lung damage and infections to 15% risk, followed by an increase of approximately 4.2 months of PFS for moving from no liver function problems to possible severe liver function problems. The MAB to move from no nausea and vomiting to severe nausea was an increase of approximately 3.7 months of PFS, and the MAB to move from no diarrhea to severe diarrhea 4 or 5 days of the month was an increase of about 2.3 months of PFS.

### Subgroup analyses

Among the six subgroups evaluated, only the differences between women based on HER2 status, whether the respondent had children, and the respondent’s country of residence were statistically significantly different using a test for the joint significance of all the interaction terms (*P* = 0.044, *P* = 0.001, and *P* = 0.001, respectively).

**Fig. 5:**
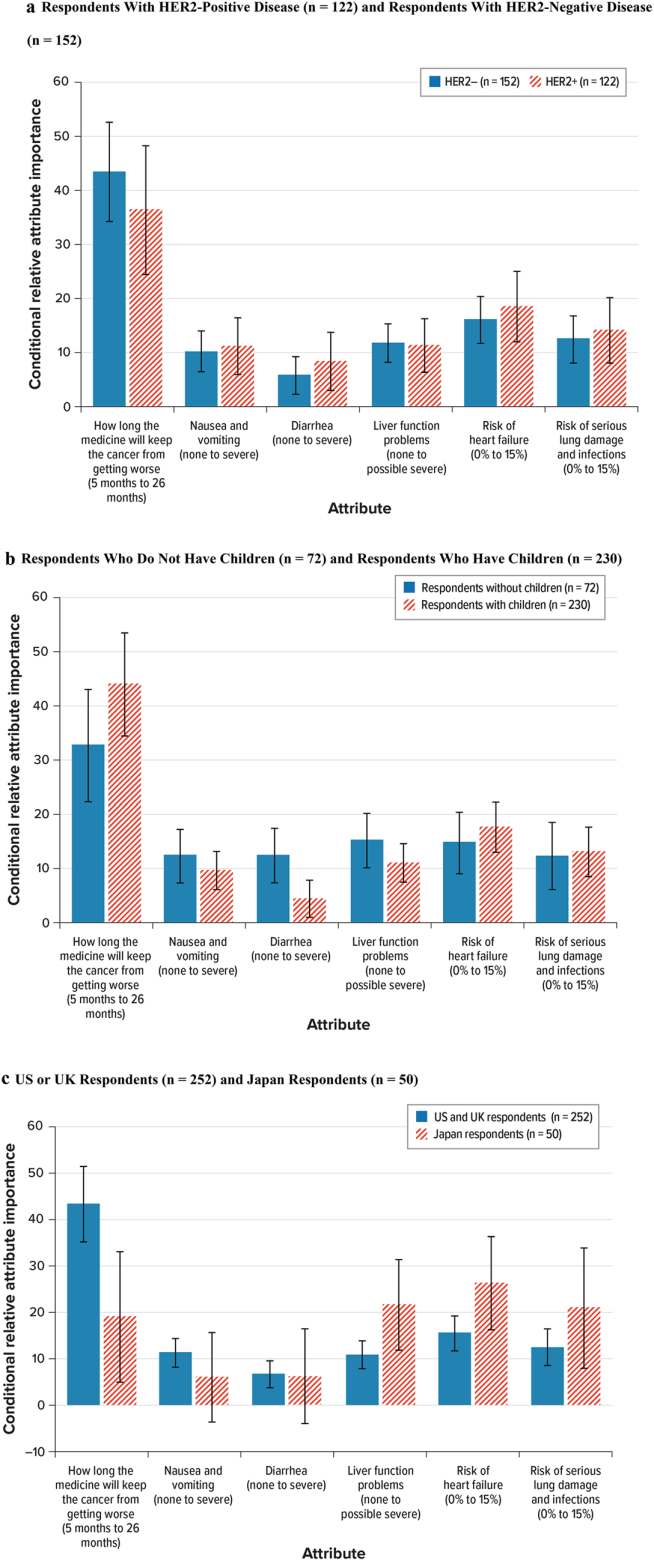
Conditional relative attribute importance: Subgroup analyses. **a** Respondents with HER2-positive disease (*n* = 122) and respondents with HER2-negative disease (*n* = 152). **b** Respondents who do not have children (*n* = 72) and respondents who have children (*n* = 230). **c** US or UK respondents (*n* = 252) and Japan respondents (*n* = 50). *HER2* human epidermal growth factor receptor 2, *UK* United Kingdom, *US* United States. Note: The vertical bars surrounding each relative importance weight estimate denote the 95% confidence interval (computed by the delta method). The bars for each subgroup are scaled so that the bar for each attribute is the percentage of the total importance of that attribute for the subgroup (scaled such that each attribute is divided by the total sum of relative attribute importance coefficients multiplied by 100 within each subgroup)

In the subgroup analysis by HER2 status, both HER2-positive respondents (*n* = 122) and HER2-negative respondents (*n* = 152) placed the most relative importance on the change in PFS from 5 to 26 months. However, additional months of PFS were more important to respondents with HER2-negative disease relative to the other attributes than for respondents with HER2-positive disease (Fig. 5a).

In the subgroup analysis by whether respondents have children, both respondents who did not have children (*n* = 72) and respondents who had children (*n* = 230) placed the most relative importance on the change in PFS from 5 to 26 months (Fig. 5b). However, relative to the other attributes, respondents with children placed more importance on increasing PFS than did respondents without children. Compared with respondents who had children, respondents without children had a stronger preference for avoiding liver function problems, diarrhea, and nausea and vomiting relative to the other attributes presented in the study.

In the subgroup analysis by country of residence, respondents in the US and UK (*n* = 252) and in Japan (*n* = 50) placed the most relative importance on the change in PFS from 5 to 26 months (Fig. 5c). However, relative to the other attributes, respondents residing in the US and UK placed more importance on increasing PFS than did respondents residing in Japan. Compared with respondents residing in the US and UK, respondents residing in Japan had a stronger preference for avoiding the risk of heart failure, liver function problems, and the risk of serious lung damage and infections relative to the other attributes presented in the study.

## Discussion

New treatments for HER2-positive metastatic breast cancer offer the possibility of better efficacy but also carry risks of different side effects than existing treatments have. This DCE study explored the importance of changes in treatment benefit (PFS) relative to treatment-related AEs (nausea and vomiting, diarrhea, the possibility of liver function problems, the risk of heart failure, and the risk of serious lung damage and infections) among women with advanced breast cancer in the US, UK, and Japan. The treatment attributes and levels were selected based on clinical outcomes and data for new and existing treatments available for HER2-positive metastatic breast cancer. The study provides insights into the willingness, on average, of women from three countries to accept AE risks in exchange for additional months of PFS and the characteristics that affected preferences in our sample.

Given the set of attributes and the range of levels for these attributes included in the survey, patients generally placed more weight on PFS gain than on the potential risk of identified AEs when deciding between hypothetical breast cancer treatments. Specifically, the change in PFS from 5 to 26 months was the most important change to respondents relative to the improvements in treatment-related AEs presented in the survey for the sample as a whole and for each of the subgroups tested. As a measure of the importance of the treatment-related AEs, we calculated the additional months of PFS required to offset a change from the best to the worst level of each of the treatment-related AEs. In general, patients required an increase of approximately 2–6 months of PFS to accept the highest level of AE risk compared with no risk. Compared with no risk, to accept a 15% risk of heart failure required the largest amount of additional months of PFS (5.8 months), followed by a 15% risk of serious lung damage and infections (4.6 months), possible severe liver function problems (4.2 months), and severe nausea and vomiting (3.7 months), although the differences were not statistically significant. Compared with no diarrhea, accepting severe diarrhea required statistically significantly fewer additional months of PFS (2.3 months) compared with accepting a 15% risk of heart failure.

In subgroup analyses, we observed that, compared with women with HER2-positive breast cancer, women with HER2-negative breast cancer placed greater weight on PFS gains relative to the other attributes, although both groups placed the greatest weight on gains in PFS relative to other attributes. There are few targeted treatments for an HER2-negative population in comparison with HER2-positive disease, where the overall prognosis is much improved in comparison with patients with HER2-negative disease. Similarly, while both groups placed the most importance on PFS gains, compared with women who do not have children, women with children placed greater priority on PFS gains relative to the other attributes. It is challenging to speculate about the drivers for these differences, but these findings suggest that HER2 status and having children may play a role in how respondents weighed additional months of PFS relative to AEs. Anecdotal evidence from the pretest interviews suggested that women with young children prioritized increased PFS. In addition, respondents in the US and UK placed greater importance on increasing PFS than did respondents residing in Japan, who had stronger preferences for avoiding AEs and AE risks. A possible explanation for this finding is that respondents in the US and UK completed the survey before the coronavirus disease 2019 (COVID-19) outbreak, while respondents in Japan completed the survey during the outbreak, potentially enhancing their concerns about AE risks. Specifically, during pretest interviews for Japan, several participants mentioned the COVID-19 virus when discussing the attribute describing the risk of serious lung infections.

According to a review of DCE studies for cancer treatment, approximately 30% of health preference studies on cancer treatment related to breast cancer [16]. Multiple DCEs have explored patients’ preferences for breast cancer treatment and their willingness to trade off side effects against other treatment outcomes and burdens in both early stage and metastatic breast cancer [28–33]. To our knowledge, this study is the first to have explored respondents’ tolerance for certain safety risks associated with HER2-targeted treatment in exchange for slowing disease progression, as well as the first study to evaluate preferences for breast cancer treatments across the US, UK, and Japan [16].

The results of this study should be interpreted in the context of several limitations. All data were self-reported. Treatment features were based on HER2-targeted treatments for metastatic breast cancer, but the sample was composed of women with stage III and IV breast cancer with any HER2 status. While we did not observe any statistically significant differences between women with stage III and women with stage IV breast cancer in their preferences for the attributes evaluated in this study, these groups may have different treatment priorities and different preferences for treatment features not evaluated. Moreover, while OS remains the gold standard for the demonstration of clinical benefit to patients, PFS was selected as the benefit attribute because the PFS data were available for the treatments of interest when the study was designed. Evaluating the importance of OS in patient preferences for HER2-targeted treatments is a direction for future research, although there is generally a lack of consistent and comparable OS data, making it a more difficult attribute to analyze. In addition, DCE surveys can include only a limited number of attributes, and factors not evaluated in the survey may further influence preferences. Although there were no missing DCE data, some individuals did not respond to all demographic questions, hence variables used to identify subgroups may be incomplete. The treatment profiles were hypothetical and may not replicate the experience of patients talking to their doctor about treatments where other considerations may come into play, such as cost or access to care. The preferences of respondents residing in the US, UK, and Japan cannot necessarily be generalized to patients in other countries, nor can the preferences of respondents with stage III or IV disease be generalized to individuals with early-stage breast cancer.

The study used a quota sample recruited from online panels and may not be representative of all women with advanced breast cancer. The final survey was administered online. Research has shown that results from online stated preference surveys are, in general, not statistically significantly different from those elicited through face-to-face interviews or mail-based surveys but yield superior response rates [34–37]. However, the online setting of the survey also may have influenced respondents’ choices. Finally, respondents in Japan completed the survey during the COVID-19 outbreak. The survey did not contain questions about COVID-19, so we could not explore the extent to which the results for the sample in Japan were affected by the COVID-19 pandemic.

## Conclusions

Preferences for HER2-targeted treatments for advanced or metastatic breast cancer among women in the US, the UK, and Japan vary by individual and disease-related characteristics. We found that patients generally valued PFS gain higher than the potential risk of identified AEs when deciding between hypothetical breast cancer treatments. The importance of increased PFS was greater for women who were HER2 negative, for those who had children, and for residents of the US and UK relative to residents of Japan. These findings provide insights into the trade-offs between slowing disease progression and treatment-related AEs that patients with breast cancer consider important for advanced or metastatic breast cancer treatments.
